# Supplementary material for: Estimation of the demand for palliative care in a hospital in southern Peru
Source: PLoS One. 2025 Mar 31;20(3):e0320228. doi: 10.1371/journal.pone.0320228 (PMC11957267; doi:10.1371/journal.pone.0320228)
Supplement: Attachment — Submitted filename: PLOS ONE peer review.docx [file pone.0320228.s001.docx]

Dear PLOS ONE editor,

Thank you for the opportunity to review this manuscript. Overall, I believe it has some interesting findings. However, there are several aspects of the methods that need clarification. Results have several limitations as well, as the data comes from a very specific population in Peru and therefore they are non-generalizable. Nevertheless, there are very few studies reporting palliative care needs in Latin America and therefore I think it might be a contribution to the field.

Find bellow some specific comments to the authors.

Introduction

Your aim only mentions the estimation of palliative care needs but not the analysis of associated factors. This should be included in the aim.

Methods

The data source, extraction procedures, and study period are not clearly explained and more detail is needed.

What was the population of the study? Did you include all patients who died in the Daniel Alcides Carrión Hospital in 2023? But you said data was collected from April to June 2024. It is not clear what that means. Is that information regarding hospital admissions? Mortality records? Why only April to June if all decedents in 2023 were included?

Later in that section, it said “The principal investigator (BMC) requested the death records for 2023 from the epidemiology department of the EsSalud Tacna Healthcare Network.”. So it is not clear if you used information from mortality records, hospital records, or both and why the years differ.

You mentioned two investigators independently extracted the variables of interest. Is that regarding hospital records? It would be useful to describe what information was extracted. If it was independently extracted, then what happened if discrepancies were found? You also said that was done anonymously. How could that have been done if you were looking at hospital records? How information from death records were linked to hospital records then?

You mentioned “incomplete medical records were excluded”. Why medical records were incomplete to the point you had to exclude them? What information was incomplete in medical records? Which proportion of all the records was that?

So, in summary, you need to clarify the population, study period,  what information was extracted and used from these two different sources and how was it linked.

In the “assessing the need for palliative care” section you describe the Rosenwax method and mention “related to one of the 10 previously established diagnoses”. You should describe what are those 10 diagnoses and if you used ICD10 codes. A table with the conditions and ICD10 codes used in the two methods would be helpful. It is also not clear if you used only the last hospital admission or all admissions in the last 12 months to derive the number of people with palliative care needs from Murtagh’s method.

The description of whether the patient had received palliative care previously is also not very clear. I understand you derived that information from hospital records, but it is unclear if you included in-patient palliative care or also palliative care in the community recorded in the hospital records. The word “consultations” is confusing as it might refer to out-patient consultations as well as hospital admissions.

It is not clear what do you mean when you said “To evaluate the factors associated with the need for palliative care, Murtagh’s method was used”. Is that the same method you describe before or something different? What about the Rosenwax method?

Which statistical software was used?

Which variables did you include in the multivariate model? And how did you decide which one to include?

How comorbidities were extracted and classified? In table 1 you describe 19 participants without comorbidities. So how did you decide when a condition was a comorbidity and when the main condition? Did you consider all chronic conditions or just a group of them?

Results

Your figure 1 is in Spanish

How was the Length of hospitalization in days for patients with palliative care needs calculated? Did you use all admissions in the last year of life or just the last one? And what is the difference between this variable and the “Hospitalization time in days”?

I do not understand this sentence “Regarding the estimation of palliative care need, it was reported that according to Murtagh et al., 90.4% of the patients required palliative care in the studied population. Concerning the estimation proposed by Rosenwax et al., 74.5% of the population needed palliative care”. Are you describing Murtagh and Rosenwax work? Or are these proportions the ones you calculated from your analysis?

Table 3: Please include the n in the first row of the columns. If I understand correctly, only 23 of the 239 participants who died in hospital had a hospital admission due to one of the 10 conditions included in Murtagh’s method. In that case, I would suggest to avoid categorising continuous variables. The sample size is too small to have 3 groups for age and length of stay. Also, why didn´t you include age and sex in the multivariate models? Those variables are likely to be important confounders in the association between palliative care needs and number of comorbidities or ED visits. Not including them in the multivariate analysis only because they are not significantly associated with your outcome variable in the univariate analysis is not a good idea. Especially if your sample size is so small.

Discussion:

In the second paragraph, you say “As expected, research using the minimum estimation according to Murtagh has found lower frequencies of palliative care need”. However, you did not use the minimum estimation from Murtagh´s method. It is not clear why you are discussing these international results then.

You discuss the differences in the estimations from the two different methods arguing the type of conditions considered. But you should also acknowledge that in Murtagh´s method, is the cause of death the one used for the estimations while in Rosenwax the cause of admission to the hospital. It would be important to discuss how this also affect the results.

You discuss the appropriateness of ICU admissions and cardiopulmonary resuscitation in the last hospital admission in your sample. However, you need to acknowledge that analysing the appropriateness of care in the general population retrospectively has limitations. Prognostication in the clinical setting is not straightforward. 36% of your sample were between 31 to 70 years old and 8% had no comorbidities. It is not surprising that a 31 years old patient without comorbidities is admitted to the ICU.

You should discuss other methods to derive palliative care needs, such as the Lancet Commission method, which is arguably more robust than Murtagh and Rosenwax ones.

You should include in your discussion the fact that you are only assessing palliative care needs among hospitalized patients. This is a strong limitation as an important proportion of the population who needs palliative care dies in the community. Also, acknowledge the fact that Murtagh´s method should include all deaths that mention any of the 10 conditions of interest, and not just people in hospital.

Also discuss the limitation of deriving comorbidities from hospital records.

In your conclusions, “We found that 90.4% and 74.5% of the deaths had a need for palliative care, according to the Murtagh and Rosenwax estimations, respectively.” You need to add, 90.4% and 74.5% of people who died in the hospital…”
