# Supplementary material for: Estimation of the demand for palliative care in a hospital in southern Peru
Source: PLoS One. 2025 Mar 31;20(3):e0320228. doi: 10.1371/journal.pone.0320228 (PMC11957267; doi:10.1371/journal.pone.0320228)
Supplement: Attachment — Submitted filename: Response to Reviewers.docx [file pone.0320228.s002.docx]

Lima, November 8, 2024

Dear Editor,

I hereby submit for your consideration the revised version of the manuscript, now titled "Estimation of the demand for palliative care in a hospital in southern Peru," for possible publication in your prestigious journal.
All co-authors have thoroughly reviewed the suggestions, comments, and annotations provided on the previous version of the manuscript, for which we received feedback on October 18 of this year. We greatly appreciate the time spent on your review and have addressed each suggestion as follows:

**Reviewer #1:**

1. **Presentation: The manuscript contains some inconsistencies that could be addressed to enhance its overall presentation, improving readability. I suggest a thorough revision to correct formatting and organizational issues**

R: Dear Reviewer, we deeply appreciate the time you have dedicated to thoroughly reading and reviewing our work. We are pleased to have your valuable observations, aimed at improving this research. Although the banner of palliative care waves fiercely around the world, in Peru this topic remains largely overlooked. Therefore, your expertise in reviewing this content is invaluable to us. Additionally, we would like to highlight that the structure of the article has been adapted in accordance with the editorial guidelines.

2. **Methodological Justification: While you have employed two well-recognized methods (Rosenwax and Murtagh) to estimate the need for palliative care, it would be valuable to include a more detailed discussion on why you selected these approaches over others reported in the literature. There are alternative methodologies that could have been considered, and addressing this would add robustness to your approach.**

R: Dear reviewer, we sincerely appreciate this brilliant observation. We have attached the added paragraph that explains the reasons behind our choice of these methods:

**<<The choice of the methods proposed by Murtagh and Rosenwax is justified by four main pillars:**

1. **Adaptability:** Their high capacity to adapt to different geographical and socioeconomic contexts was essential for considering the local factors that influence the demand for palliative care (4). In contrast, other methods, such as the one proposed by Gómez-Batiste, exhibit less flexibility in adjusting to various local realities (15).
2. **Data integration:** They facilitate the incorporation of data from multiple sources, which improves the accuracy of estimates and provides a more comprehensive view of patients' needs (15).
3. **Robustness and empirical validation:** Their robustness and empirical validation, demonstrated by Jeba et al., ensure the reliability of the method (16). This surpasses other approaches, such as the one proposed by Howard et al., which do not provide long-term projections (17).
4. **Flexibility in the incorporation of factors:** The method's ability to integrate new weighting factors, such as those proposed by the Lancet Commission, allows estimates to be dynamically updated without compromising the analysis (4). **>>**
3. **Prevalent Population: It would be interesting to complement your methodology by considering the prevalent population living with the disease and eligible for palliative care, rather than relying solely on death records. This could provide a more comprehensive view of the potential demand.**

R: Dear reviewer, thank you for your valuable observation. However, it is important to note that the hospital where this study was conducted does not have a systematic registry of the prevalence of chronic diseases. This is due to structural limitations within the healthcare system, such as a lack of resources to implement epidemiological surveillance systems and longitudinal data registries. This deficiency complicates health service planning and the accurate assessment of the potential demand for palliative care.

4. **The use of Poisson regression with robust variance to calculate prevalence ratios (PR) is appropriate for the data, given the high prevalence of palliative care needs in the population. However, it would be beneficial to provide a more detailed justification for choosing this method over alternatives such as logistic regression, which is commonly used in prevalence studies. Additionally, while the adjustment for multiple variables in the multivariable model is commendable, a more thorough discussion regarding the selection of these variables would strengthen the analysis. Specifically, explaining why certain variables were chosen a priori and included in the adjusted model only if they met the p-value threshold of <0.05 in the crude model would provide greater clarity.**

R: I appreciate your observation regarding the use of logistic regression. It is true that, in our study, the results show a high prevalence of palliative care needs, reaching up to 90.4% according to the estimates from Murtagh's method. In contexts of high prevalence, logistic regression tends to overestimate odds ratios, which can result in less intuitive and less useful estimates for clinical interpretation.

For this reason, we opted to use Poisson regression with robust variance. This approach provides more precise and direct estimates of prevalence ratios, which are easier to interpret in clinical terms. This is especially relevant when describing the association between multiple comorbidities and the need for palliative care in populations with a high disease burden.

A threshold of p < 0.05 was established to filter the variables that presented sufficient statistical evidence to be included in the adjusted model. It is worth mentioning that, up to the time of the review, no studies have been identified that associate the estimation of the need for palliative care with other variables. Therefore, the criterion of selecting variables based on a preexisting theory was not employed. The statistical analysis section was adapted in the following manner:

**<< The analysis was performed using Stata V17. The variables were described in terms of frequencies, percentages, measures of central tendency, and dispersion. To evaluate the factors associated with the need for palliative care, Murtagh’s method was used, as different studies highlight that this method provides the most inclusive estimates regarding the extent of palliative care needs in the population** [**(7,13,15)**](https://www.zotero.org/google-docs/?bggUam)**. Poisson regression models were used to calculate prevalence ratios (PR) with their respective 95% confidence intervals (CI95%). This method was preferred over logistic regression due to the high prevalence of observed palliative care needs. In high-prevalence contexts, logistic regression tends to overestimate odds ratios, which can result in less intuitive and less useful estimates for clinical interpretation. Therefore, Poisson regression with robust variance was chosen, as this approach provides more accurate and direct estimates of prevalence ratios, facilitating their interpretation in clinical terms. This is particularly relevant when describing the association between multiple comorbidities and the need for palliative care in populations with a high disease burden.**

**Variables with a p-value < 0.05 in the crude model were included in the adjusted model, using this threshold to select those with sufficient statistical evidence. It is important to note that, as of the time of review, no studies have been identified that link the estimation of palliative care needs with other variables. Therefore, a variable selection criterion based on preexisting theory was not applied.>>**

5. **Figure 1: This figure is in Spanish. Since the publication is in English, I suggest translating it to maintain consistency throughout the document.
   Table 3: Regarding Table 3, could you clarify what "REF" refers to? A brief explanation would help readers understand this more clearly.**

R: Thank you for your observation. The translation of Figure 1 has been completed. Additionally, it has been specified in the table footnote that the term "REF" stands for Reference Value.

6. **Regional Comparison: I suggest comparing your estimates with studies from other regions. For instance, Chile has two studies analyzing the demand and need for palliative care, which could serve as useful references for comparison.**

R:	Thank you for your contribution. We have conducted the search based on your suggestion and have generated a new paragraph regarding the comparison in the regional context, in this case, Latin America. Below is the paragraph:

**<< In the Latin American context, there is currently limited literature estimating the need for palliative care at both hospital and national levels. In Chile, two studies have been published that assess the demand for palliative care based on the proportion of deceased patients requiring such services, taking into account the burden of severe health-related suffering. This methodology aligns with the recommendations of the Lancet Commission report (4). The first study, which applied this methodology to patients who died between 2018 and 2020, found that 65% required palliative care (18). The second study, encompassing a broader population (deaths from 1997 to 2019), reported that the need for palliative care was 44.5% in 1997 and increased to 48% in 2019 (19). Additionally, it projects that this need will rise from 58,000 patients in 2021 to 105,000 by 2050. In Brazil, a study analyzing death records of cancer patients from 2008 to 2014, utilizing the World Health Organization’s model to estimate palliative care needs, found that 80% of terminal cancer patients required these services (20). >>**

**Reviewer #2:**

Dear reviewer, your observations have been incredibly enlightening and have undoubtedly strengthened this work, which, despite its limitations, aims to broaden the perspective on palliative care in the country.

1. Introduction

Your aim only mentions the estimation of palliative care needs but not the analysis of

associated factors. This should be included in the aim.

R: The objective has been modified as evidenced in the following sentence:

<< Therefore, the objective of this study is to estimate the need for palliative care and its associated factors in patients who died at the Daniel Alcides Carrion Hospital (Tacna, Peru) during 2023.>>

2. Methods

**The data source, extraction procedures, and study period are not clearly explained and more detail is needed. What was the population of the study? Did you include all patients who died in the Daniel Alcides Carrión Hospital in 2023? But you said data was collected from April to June 2024. It is not clear what that means. Is that information regarding hospital admissions? Mortality records? Why only April to June if all decedents in 2023 were included? Later in that section, it said “The principal investigator (BMC) requested the death records for 2023 from the epidemiology department of the EsSalud Tacna Healthcare Network.”. So it is not clear if you used information from mortality records, hospital records, or both and why the years differ. You mentioned two investigators independently extracted the variables of interest. Is that regarding hospital records? It would be useful to describe what information was extracted. If it was independently extracted, then what happened if discrepancies were found? You also said that was done anonymously. How could that have been done if you were looking at hospital records? How information from death records were linked to hospital records then? You mentioned “incomplete medical records were excluded”. Why medical records were incomplete to the point you had to exclude them? What information was incomplete in medical records? Which proportion of all the records was that? So, in summary, you need to clarify the population, study period, what information was extracted and used from these two different sources and how was it linked.**

R: << Analytical cross-sectional study. Data were collected from death records of patients who died during hospitalization across all departments at the Daniel Alcides Carrión Hospital (Tacna, Peru) in 2023. Adult patients (over 18 years old) were included, and those whose identification documents did not match the death records were excluded.

The principal investigator (BMC) requested from the epidemiology department of the EsSalud Tacna Healthcare Network a list of deaths that occurred during hospitalization in 2023 at the Daniel Alcides Carrión Hospital, located in the city of Tacna. This list was provided in Excel format, with deaths numbered according to their order of occurrence and including the national identity document number to verify the information. Subsequently, access was granted to the EsSalud Intelligent Health Service (ESSI), which allows for consultation of the patient’s electronic medical record, containing all medical care received, both in outpatient settings and during hospitalization. Access to this information was made using the national identity document number.

Two investigators (BMC and WZR) independently reviewed the electronic medical records of deceased patients and extracted the variables of interest into an Excel spreadsheet. Discrepancies identified during the comparison of the two extractions were resolved with the assistance of a third author (MHZ). >>

**In the “assessing the need for palliative care” section you describe the Rosenwax method and**

**mention “related to one of the 10 previously established diagnoses”. You should describe what**

**are those 10 diagnoses and if you used ICD10 codes. A table with the conditions and ICD10**

**codes used in the two methods would be helpful. It is also not clear if you used only the last**

**hospital admission or all admissions in the last 12 months to derive the number of people with**

**palliative care needs from Murtagh’s method.**

R: Supplementary tables related to the Rosenwax and Murtagh methods have been added as supplementary material, as shown below:

Supplementary Material:

AGREGAR TABLA AQUÍ (PABLO)

All hospital admissions made in the last 12 months were considered to evaluate whether the cause of death had also been, at any time, the cause of a previous hospitalization within that time period.

**Which statistical software was used?**

R: <<The analysis was performed using Stata V17.>>

**Which variables did you include in the multivariate model? And how did you decide which one**

**to include?**

R: <<Variables with a p-value < 0.05 in the crude model were included in the adjusted model, using this threshold to select those with sufficient statistical evidence. It is important to note that, as of the time of review, no studies have been identified that link the estimation of palliative care needs with other variables. Therefore, a variable selection criterion based on preexisting theory was not applied. >>

**How comorbidities were extracted and classified? In table 1 you describe 19 participants**

**without comorbidities. So how did you decide when a condition was a comorbidity and when**

**the main condition? Did you consider all chronic conditions or just a group of them?**

R: Regarding comorbidities, all chronic diseases that the patient had were included in this group. On the other hand, the group of 19 individuals "Without comorbidities" refers to those who may have died due to traffic accidents, poisoning, or an acute illness. Additionally, the primary condition was defined as the disease that was the direct cause of the patient's death, as recorded in the electronic medical record.

Results

Your figure 1 is in Spanish

R: The figure has been translated into English.

PEGAR FIGURA

**How was the Length of hospitalization in days for patients with palliative care needs calculated? Did you use all admissions in the last year of life or just the last one? And what is the difference between this variable and the “Hospitalization time in days”?**

There has been an error on our part when drafting this section. We considered the variable “Duration of hospitalization” as the days that patients with palliative care needs were hospitalized during their last hospitalization. This has been corrected in the following paragraph:

<< Other variables were also included, such as age, sex, duration of the last hospitalization in days, the acute resuscitation plan (use of resuscitation measures in a patient with significant clinical deterioration), the number of comorbidities, and variables related to the use of services and procedures in patients who may have needed palliative care. >>

Additionally, the name of the variable has been changed in Table 01.

**I do not understand this sentence “Regarding the estimation of palliative care need, it was reported that according to Murtagh et al., 90.4% of the patients required palliative care in the studied population. Concerning the estimation proposed by Rosenwax et al., 74.5% of the population needed palliative care”. Are you describing Murtagh and Rosenwax work? Or are these proportions the ones you calculated from your analysis?**

R: The formulation of the following sentence has been changed.

<<Regarding the estimation of palliative care needs, by applying the method proposed by Murtagh et al. to our population, it was found that 90.4% of the patients required palliative care. On the other hand, using the estimation proposed by Rosenwax et al., it was determined that 74.5% of the population needed palliative care.>>

**Table 3: Please include the n in the first row of the columns. If I understand correctly, only 23**

**of the 239 participants who died in hospital had a hospital admission due to one of the 10**

**conditions included in Murtagh’s method. In that case, I would suggest to avoid categorising**

**continuous variables. The sample size is too small to have 3 groups for age and length of stay.**

**Also, why didn´t you include age and sex in the multivariate models? Those variables are likely**

**to be important confounders in the association between palliative care needs and number of**

**comorbidities or ED visits. Not including them in the multivariate analysis only because they**

**are not significantly associated with your outcome variable in the univariate analysis is not a**

**good idea. Especially if your sample size is so small.**

R: The table was modified according to your suggestions.

**Table 03: Factors Associated with the Need for Palliative Care in the Studied Population (n=239)**

| **Variables** | **No palliative care needed n (%)** | **Palliative care needed n (%)** | **Crude PR (95% CI)*** | **Adjusted PR (95% CI)** |  |
| --- | --- | --- | --- | --- | --- |
|  |  |  |  |  |  |
| Age in years | 73 (36 to 97) | 76 (31 to 98) | 1.00 (0.99 to 1.00) | 1.00 (0.99 to 1.00) |  |
| Sex |  |  |  |  |  |
| Female | 14 (13.0) | 94 (87.0) | REF | REF |  |
| Male | 9 (6.9) | 122 (93.1) | 1.03 (0.99 to 1.08) | 1.04 (1.00 to 1.08) |  |
| Length of hospitalization | 7 (1 a 45) | 9 (1 a 88) | 1.00 (0.99 to 1.00) |  |  |
| Acute resuscitation plan |  |  |  |  |  |
| Yes | 9 (12.7) | 62 (87.3) | REF |  |  |
| No | 14 (8.4) | 152 (91.6) | 1.02 (0.97 to 1.07) |  |  |
| Number of comorbidities |  |  |  |  |  |
| None | 9 (47.4) | 10 (52.6) | REF | REF |  |
| One | 5 (6.2) | 76 (93.8) | 1.27 (1.09 to 1.48) | 1.22 (1.06 to 1.41) |  |
| Two or more | 9 (6.5) | 130 (93.5) | 1.27 (1.09 to 1.47) | 1.21 (1.05 to 1.39) |  |
| Emergency department visits in the last 6 months* |  |  |  |  |  |
| None | 10 (20.8) | 38 (79.2) | REF | REF |  |
| One | 5 (8.8) | 52 (91.2) | 1.07 (0.99 to 1.15) | 1.03 (0.97 to 1.10) |  |
| Two or more | 8 (6.0) | 126 (94.0) | 1.08 (1.01 to 1.16) | 1.03 (0.98 to 1.09) |  |
| Outpatient visits in the last 6 months* |  |  |  |  |  |
| None | 9 (34.6) | 17 (65.4) | REF | REF |  |
| One | 1 (7.7) | 12 (92.3) | 1.16 (1.02 to 1.33) | 1.11 (0.97 to 1.25) |  |
| Two or more | 13 (6.5) | 186 (93.5) | 1.17 (1.05 to 1.31) | 1.10 (0.99 to 1.22) |  |
| **Last hospitalization** |  |  |  |  |  |
| ICU admission |  |  |  |  |  |
| Yes | 9 (12.7) | 62 (87.3) | 0.98 (0.93 to 1.02) |  |  |
| No | 14 (8.4) | 152 (91.6) | REF |  |  |
| ICU stay duration in days | 3 (1 to 45) | 10 (1 to 146) | 1.00 (0.99 to 1.00) |  |  |

REF: Reference value

*Simple Poisson regression with robust variance

**Multiple Poisson regression with robust variance; a model was generated with the variables: number of comorbidities, emergency visits in the last 6 months, and outpatient visits in the last 6 months.

Discussion

**In the second paragraph, you say “As expected, research using the minimum estimation**

**according to Murtagh has found lower frequencies of palliative care need”. However, you did**

**not use the minimum estimation from Murtagh´s method. It is not clear why you are**

**discussing these international results then.**

R: Thank you for your observation. It is important to note that the comparison was made using the minimum estimation, as most studies employing Murtagh's proposed method have followed this approach. Therefore, these studies were mentioned to provide a point of reference.

**You discuss the differences in the estimations from the two different methods arguing the type**

**of conditions considered. But you should also acknowledge that in Murtagh´s method, is the cause of death the one used for the estimations while in Rosenwax the cause of admission to the hospital. It would be important to discuss how this also affect the results.**

R: Thank you for this contribution. A sentence addressing your suggestion was included, which is highlighted at the end of the paragraph.

<<It is important to highlight a significant variation in our study regarding the estimations of Murtagh (90.4%) and Rosenwax (74.5%). This finding is consistent with two previous studies conducted between 2010 [(7)](https://www.zotero.org/google-docs/?KL8NBr) and 2013 [(13)](https://www.zotero.org/google-docs/?u6nbci), which reported palliative care estimates according to Murtagh at 74% and 78%, and according to Rosenwax at 38% and 40.7%, respectively. The difference between one estimation and another lies in the diagnoses included in each method. Murtagh considers, in addition to the diagnoses shared with Rosenwax, additional conditions such as breast, colorectal, lung, and prostate cancers, as well as cerebrovascular diseases, chronic renal insufficiency, liver diseases, and respiratory diseases. Therefore, the discrepancy in percentages may reflect the different definitions and criteria used in each method to assess the need for palliative care, highlighting the importance of considering multiple approaches to obtain a comprehensive view of palliative care needs in different populations and contexts. **It is important to mention that the method proposed by Murtagh is based on the cause of death for estimation, whereas the Rosenwax method uses the cause of hospital admission. These methodological differences can lead to divergent estimations, which may underestimate or overestimate the need for palliative care depending on the available data and the context in which they are applied.>>**

**You discuss the appropriateness of ICU admissions and cardiopulmonary resuscitation in the**

**last hospital admission in your sample. However, you need to acknowledge that analysing**

**the appropriateness of care in the general population retrospectively has limitations.**

**Prognostication in the clinical setting is not straightforward. 36% of your sample were**

**between 31 to 70 years old and 8% had no comorbidities. It is not surprising that a 31 years old**

**patient without comorbidities is admitted to the ICU.**

R: Thank you for your excellent observation. A sentence based on your suggestion was added at the end of the paragraph.

<<We found that, during their last hospitalization, 29.3% of the deceased were admitted to the ICU, and 27.8% had an acute resuscitation plan that involved receiving cardiopulmonary resuscitation or the administration of vasopressors. It would be of interest for future studies to assess whether these procedures were beneficial. In this regard, a systematic review, which included studies from 1995 to 2015, concluded that 30% of patients nearing death underwent unnecessary procedures [(29)](https://www.zotero.org/google-docs/?EgVooM). Finally, this scenario could be attributed to a combination of the culture of 'doing everything possible,' the fear of death, and a false sense of hope from healthcare personnel or the patient's family members. This leads to a series of repercussions, both financially for the healthcare system and, most importantly, in the disregard for the patient's dignity and quality of life. **It is important to mention that, although 36% of the patients were between 31 and 70 years old and 8% had no comorbidities, the analysis does not fully capture the clinical reasons that justified their admission to the ICU. In the case of young patients without comorbidities, these decisions are often considered appropriate due to their higher likelihood of recovery.>>**

**You should discuss other methods to derive palliative care needs, such as the Lancet**

**Commission method, which is arguably more robust than Murtagh and Rosenwax ones.**

R: Dear reviewer, while the method proposed by The Lancet is considered the most robust for estimating the need for palliative care, the authors of this work chose not to replicate it due to the statistical complexity its implementation might entail. Nevertheless, this opens the door for future research to undertake such an analysis.

**You should include in your discussion the fact that you are only assessing palliative care needs**

**among hospitalized patients. This is a strong limitation as an important proportion of the**

**population who needs palliative care dies in the community. Also, acknowledge the fact that**

**Murtagh´s method should include all deaths that mention any of the 10 conditions of interest,**

**and not just people in hospital.**

**Also discuss the limitation of deriving comorbidities from hospital records.**

R: Dear reviewer, thank you for your observation. This suggestion was added to the limitations paragraph, as evidenced in the following:

**This was conducted in a hospital that provides services to a specific population within Peru, with its own characteristics and trends. Additionally, there is a possibility that the data collected from death records is underreported, especially regarding neurological diseases such as dementia and Parkinson's. Likewise, our estimation, being based on the primary diagnosis of the cause of death, could underestimate the need for palliative care, as comorbidities, which could increase the demand for these services, are not fully considered. Furthermore, this study only assessed palliative care needs among hospitalized patients, which is a significant limitation as a large proportion of individuals requiring palliative care die in the community. The method used (Murtagh) should account for all deaths mentioning any of the 10 conditions of interest, not just those occurring in hospitals.**

**R: It was added based on your suggestion.**

We found that 90.4% and 74.5% of hospital deaths had a need for palliative care, according to the Murtagh and Rosenwax estimations, respectively. The diagnoses that most contributed to these estimations were malignant neoplasms, chronic obstructive pulmonary disease, and renal and hepatic insufficiencies. The need for palliative care was associated with having more comorbidities and having had an outpatient visit in the last six months.
