# Supplementary material for: Estimation of the demand for palliative care in a hospital in southern Peru
Source: PLoS One. 2025 Mar 31;20(3):e0320228. doi: 10.1371/journal.pone.0320228 (PMC11957267; doi:10.1371/journal.pone.0320228)
Supplement: Attachment — Submitted filename: Response to Reviewers.2.0docx.docx [file pone.0320228.s003.docx]

Lima, January 26, 2025

Dear Editor,

I hereby submit for your consideration the revised version of the manuscript, now titled "Estimation of the demand for palliative care in a hospital in southern Peru," for possible publication in your prestigious journal.
All co-authors have thoroughly reviewed the suggestions, comments, and annotations provided on the previous version of the manuscript, for which we received feedback on October 18 of this year. We greatly appreciate the time spent on your review and have addressed each suggestion as follows:

**Reviewer #1:**

1. Justification of Methods Selection: Although the selection of the Rosenwax and Murtagh methods is well justified, a brief discussion on why alternative methods, such as the Lancet Commission approach, were not considered would further strengthen the methodological rationale.

R: Dear Reviewer, we appreciate your valuable observation. We have included a paragraph explaining why the approach proposed by the Lancet Commission was not used:

<< We chose not to use the Lancet Commission approach, although we recognize its relevance, for two reasons: (1) the methods proposed by Rosenwax and Murtagh are better suited for analyzing hospital deaths and specific diagnoses, which are directly applicable to our population, whereas the Lancet Commission approach is more appropriate for regional or national-level estimations; (2) the methods proposed by Rosenwax and Murtagh methods ensure greater comparability with previous studies conducted in similar contexts and significantly contribute to the literature in low- and middle-income countries. >>

2. The authors opted for the “medium” category in the Rosenwax method and “high medium” in the Murtagh method. While reasonable, a more explicit discussion of how these choices may influence the results compared to the minimum or maximum categories would improve the manuscript.

R: Dear reviewer, thank you for your valuable feedback. We clarify the reasons behind selecting the "medium" category in the Rosenwax method and the "high-medium" category in the Murtagh method for this study. The "medium" category in the Rosenwax method allows for the inclusion of hospitalizations related to key diagnoses within the past 12 months, avoiding both the underestimation of patients who may require palliative care (as in the "minimum" category) and the overestimation of irrelevant cases (as in the "maximum" category). Similarly, the "high-medium" category in the Murtagh method incorporates emerging conditions and contributing diagnoses, ensuring more precise estimates aligned with previous studies, without resorting to excessive generalizations. However, we believe that including this level of detail in the manuscript might overwhelm the reader with additional information that could be unnecessary.

3. Limited Discussion of Methodological Alternatives: The authors emphasize the adaptability and robustness of their selected methods but could briefly discuss the limitations of other approaches (e.g., Gómez-Batiste or Howard et al.) to provide a more balanced perspective.

R: Dear reviewer, thank you for your comment. Regarding the limitations of other approaches, these have been addressed in the methodology section, where the selection of the methods proposed by Rosenwax and Murtagh is also justified. For this reason, we believe it is not necessary to reiterate them in the discussion.
